# Aetiology of auditory dysfunction in amusia: a systematic review

**DOI:** 10.1186/1755-7682-6-16

**Published:** 2013-04-24

**Authors:** Daniel AJ Casey

**Affiliations:** 1 Merton College, University of Oxford, Oxford OX1 4JD, UK

**Keywords:** Amusia, Aetiology, Auditory agnosia, Otology, Neurology

## Abstract

**Background:**

Amusia, a music-specific agnosia, is a disorder of pitch interval analysis and pitch direction change recognition which results in a deficit in musical ability. The full range of aetiological factors which cause this condition is unknown, as is each cause’s frequency. The objective of this study was to identify all causes of amusia, and to measure each of their frequencies.

**Methods:**

Design: systematic review was conducted by search of multiple databases for articles related to the aetiology of amusic auditory dysfunction. The Preferred Reporting Items for Systematic Reviews and Meta-Analyses (PRISMA) guidelines for reporting of systematic reviews were followed, utilizing the PRISMA checklist and PRISMA flowchart methodology. Setting: Retrospective medical database review. Main outcome measures: evidence yielded from the systematic review process.

**Results:**

The initial search protocol identified 5723 articles. Application of a classification review filter and exclusion of irrelevant or duplicates led to the initial identification of 56 relevant studies which detailed 301 patients. However, these studies were of poor quality. Because of this, synthesis and statistical analysis were not appropriate.

**Conclusion:**

Although initially a large number of relevant studies were identified, and might point in future to potential diagnostic categories, it was not appropriate to synthesise and analyse them due to poor quality, considerable heterogeneity and small numbers. This suggests that large, high quality studies focussed directly on understanding the aetiology of amusia are required.

## Background

Amusia is a music-specific auditory agnosia consisting of neurological deficit in musical ability [1]. Knoblauch’s definition is ‘the loss of a musical ability, such as the comprehension of music, the production of music, or the ability to read or write musical notation’ [2].

However, a grey area lies between ‘amusia’ and auditory agnosias which impair auditory function related to pitch processing, interval analysis, timbre, rhythm or the emotional components of music but with sparing of gross musical ability [3]. This study will focus on studies in which there is a clear loss of musical ability and thus satisfy Knoblauch’s definition.

Fry estimates that 4.2% of the UK adult population may be amusic [4]. As music and melody form a fundamental part of human experience, amusia is associated with significant distress, especially for musical professionals. Removal of a stimulus which in some instances can activate neuroanatomical regions associated with intense pleasure can represent a severe handicap [5].

Thus, a systematic examination of the aetiology of amusia may be of considerable importance in forming a differential diagnosis in the clinic and in informing prevention strategies. Understanding the aetiological factors behind amusia can also shed light on musical auditory function and aid in deficit prediction. The neural basis of music is a matter of intense investigation.

However, no such aetiological categorization has yet been conducted. Here, a systematic review of the causes of amusia is performed.

## Method

This systematic review followed the Preferred Reporting Items for Systematic Reviews and Meta-Analyses (PRISMA) guidelines for reporting of systematic reviews, utilizing the PRISMA checklist and PRISMA flowchart methodology [6].

### Initial search protocol

Systematic review was conducted by search of multiple databases for articles related to the aetiology of amusic auditory dysfunction (Medline and The Cochrane Library). Key Terms used to identify the concept of amusic auditory dysfunction included amusia (sensory and motor), dysmusia, tone deaf(ness), tune deaf(ness), pitch processing deficit and note deaf(ness). Keywords used to identify aetiological frequency included aetiology, etiology, aetiologic(al) factor(s), aetiol*, etiol*, pathophysiology identification, risk factor(s) and cause(s). Aetiological keywords were combined with those specific to amusic auditory dysfunction. A secondary search was then conducted, and the grey literature was also searched.

### Filter procedure

Initial search using the amusic key-terms, with and without aetiological key-term modifiers, yielded 5723 articles for assessment. Articles to form part of the systematic review were then filtered based on a classification review filter. Studies to be included had to be directly relevant to amusia, contain a definition of amusia consistent with the current literature (i.e. music specific auditory agnosia), and provide a full aetiological explanation for all subjects with accompanying diagnostic evidence.

After elimination of irrelevant articles and application of the above criteria, results were narrowed to 56 papers containing 301 patients ranging from 1878 to 2012, including articles in English, German, Spanish, French, Italian, Russian and Japanese (non-English language articles were translated into English).

However, 40 of these 56 were single case reports; the remaining studies also contained small patient numbers (and/or had amusia as a secondary concern within the paper). This meant that the quality of evidence found was very poor. Due to this, no studies found were appropriate for synthesis and analysis.

## Results

**Figure 1:**
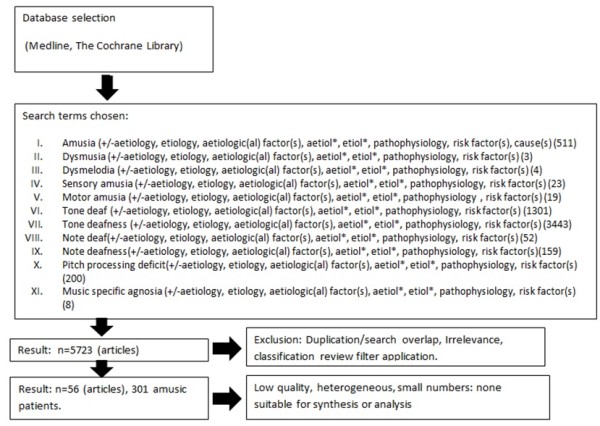
Article selection process following PRISMA guidelines.

The PRISMA flow diagram (Figure 1) summarizes the article selection process. The initial search protocol identified 5723 articles. Application of the classification review filter and exclusion of irrelevant or duplicates led to the initial identification of 56 relevant studies [7-62]. These studies contained 301 patients. Articles had been published from 1878 to 2012 and included papers in English, German, Spanish, Italian, French, Russian and Japanese (non-English language articles were translated into English).

However, none were suitable for synthesis and further analysis because they were poor quality with regards to small numbers. In addition, considerable heterogeneity precluded synthesis.

## Discussion

Although initially a large number of relevant studies were identified, and might point in future to potential diagnostic categories, it was not possible to synthesise them due to poor quality, considerable heterogeneity and small numbers.

40 of the 56 initially identified studies were single case reports: clearly, drawing conclusions from a synthesis of these would not be appropriate. The remaining studies also contained relatively small patient numbers. In addition, these studies were extremely heterogeneous. Studies came from a wide range of time periods, with different reporting methods.

On this basis, to answer the question of amusia aetiology, it is necessary in the future to design and implement large, high quality studies with agreed diagnostic criteria.

### Implications for research and future hypotheses

Understanding and quantifying amusia aetiology would be useful for assessment of this condition in a clinical setting. This paper suggests that this will not be possible until relevant high quality evidence is produced.

**Figure 2:**
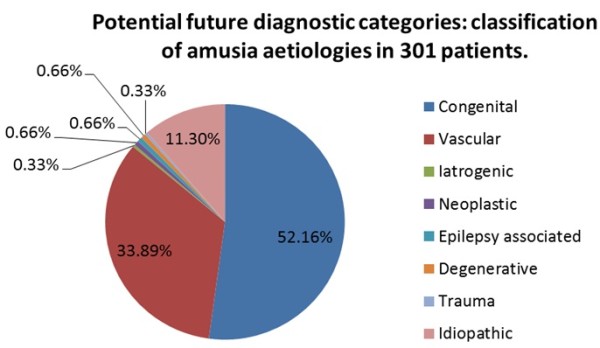
**Potential future diagnostic categories: classification of amusia aetiologies in 301 patients.** This figure is simply a record of the aetiologies identified in studies which were not suitable for analysis or synthesis but nonetheless contained amusic patients. Speculatively, some of these aetiological categories might be important when a full systematic review is possible in the future.

Before this evidence becomes available, it is interesting to think of the possible diagnostic categories which might emerge – classification of the 56 ‘low quality’ (with respect to performing a systematic review) studies identified above suggests the following possibilities: vascular, congenital, iatrogenic, neoplastic, epilepsy-associated, degenerative, traumatic and idiopathic (Figure 2). Of course, which of these possibilities, if any, are relevant, awaits further investigation.

**Figure 3:**
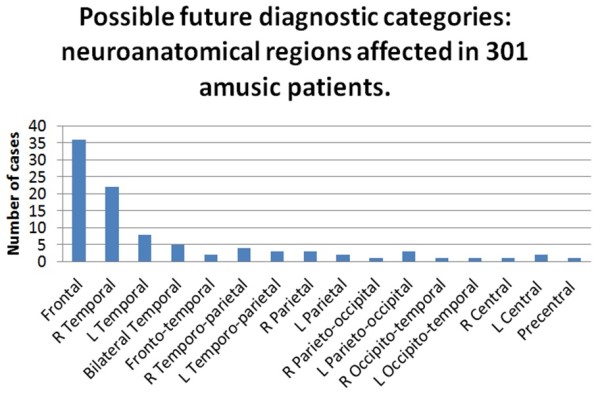
Possible future diagnostic categories: neuroanatomical sites involved in 301 amusic cases.

We might hypothesize that some cause of neural damage to key brain regions is required for the onset of amusia. Although it is inappropriate to draw any type of quantitative conclusion from the initial 56 studies identified above, categorization of the neuroanatomical areas involved suggests a host of possible key areas (Figure 3). However, this is entirely speculative, and many of these possibilities may not be relevant. Hypothetically, the underlying commonality of any aetiological cause of amusia might be temporal lobe damage to a putative ‘pitch centre’ (or, at least, a key region involved in pitch processing) in lateral Heschl’s gyrus (HG) [63].

## Conclusion

In conclusion, due to the infrequent and irregular nature of the evidence related to amusia aetiology, all relevant studies suffer from small numbers and considerable heterogeneity. This, in addition to the other problems outlined above, suggests that large, high quality studies focussed directly on understanding the aetiology of amusia are required before a synthesis and analysis can take place.

## Competing interests

The author declare that he has no competing interests.

## Authors’ contributions

DC was the sole author.
